# High Specificity of Single Inertial Sensor-Supplemented Timed Up and Go Test for Assessing Fall Risk in Elderly Nursing Home Residents

**DOI:** 10.3390/s22062339

**Published:** 2022-03-17

**Authors:** Frédéric Dierick, Pierre-Loup Stoffel, Gaston Schütz, Fabien Buisseret

**Affiliations:** 1 Laboratoire d’Analyse du Mouvement et de la Posture (LAMP), Centre National de Rééducation Fonctionnelle et de Réadaptation—Rehazenter, Rue André Vésale 1, 2674 Luxembourg, Luxembourg; frederic.dierick@rehazenter.lu; 2 Faculté des Sciences de la Motricité, Université Catholique de Louvain, Place Pierre de Coubertin 2, 1348 Ottignies-Louvain-la-Neuve, Belgium; 3 CeREF-Technique, Chaussée de Binche 159, 7000 Mons, Belgium; 4 Forme and Fonctionnement Humain Laboratory, Department of Physical Therapy, Haute Ecole Louvain en Hainaut, Rue Trieu Kaisin 136, 6061 Montignies-sur-Sambre, Belgium; pierreloupstoffel@gmail.com; 5 Centre National de Rééducation Fonctionnelle et de Réadaptation—Rehazenter, Rue André Vésale 1, 2674 Luxembourg, Luxembourg; gaston.schuetz@rehazenter.lu; 6 Service de Physique Nucléaire et Subnucléaire, UMONS Research Institute for Complex Systems, Université de Mons, Place du Parc 20, 7000 Mons, Belgium

**Keywords:** TUG, kinematics, fall risk, logistic regression, elderly, inertial sensor

## Abstract

The Timed Up and Go test (TUG) is commonly used to estimate the fall risk in the elderly. Several ways to improve the predictive accuracy of TUG (cameras, multiple sensors, other clinical tests) have already been proposed. Here, we added a single wearable inertial measurement unit (IMU) to capture the residents’ body center-of-mass kinematics in view of improving TUG’s predictive accuracy. The aim is to find out which kinematic variables and residents’ characteristics are relevant for distinguishing faller from non-faller patients. Data were collected in 73 nursing home residents with the IMU placed on the lower back. Acceleration and angular velocity time series were analyzed during different subtasks of the TUG. Multiple logistic regressions showed that total time required, maximum angular velocity at the first half-turn, gender, and use of a walking aid were the parameters leading to the best predictive abilities of fall risk. The predictive accuracy of the proposed new test, called i + TUG, reached a value of 74.0%, with a specificity of 95.9% and a sensitivity of 29.2%. By adding a single wearable IMU to TUG, an accurate and highly specific test is therefore obtained. This method is quick, easy to perform and inexpensive. We recommend to integrate it into daily clinical practice in nursing homes.

## 1. Introduction

The proportion of elderly people is steadily increasing. According to the World Health Organization (WHO) estimates, it will account for 22% of the world’s population by 2050. Physiological changes caused by ageing result in deterioration of balance, coordination, and strength, leading to an increased incidence of falls in people over 65 years. Falls are one of the top five causes of death in this age group and their incidence is particularly increasing in people living in nursing homes, with an average of 1.7 falls per bed per year compared to 0.65 in people living independently [1]. In addition, falls lead to more complications in people living in nursing homes, with 10 to 25% of falls resulting in a fracture or open wound [2]. The main risk factors for falls are muscle weakness, balance problems, and gait disturbances [2].

The Timed Up and Go test (TUG) [3] is commonly used in the medical field to predict fall risk in the elderly. Very simply, TUG consists of measuring the time it takes a person to get up from a chair with arms after having heard a “Go”, walk forward three meters at a comfortable pace, turn around, walk back to the chair, and sit down again. According to the pioneering study [4], a time greater than 14.0 s indicates a potential fall risk. Obvious advantages of the TUG are the following. It is quick to perform, it requires no equipment other than a chair and a stopwatch, and it involves a sequence of movements common to daily life: getting up, walking, turning, and sitting. In the review [5], it is concluded that the predictive power of the TUG is greater in people living in institutions than in people living independently. This conclusion is shared by [6]. However, this test is limited by the fact that total time is the only parameter measured and that it categorizes fall risk according to a threshold value that is increasingly controversial in the literature. The threshold used to identify fallers in nursing homes actually varies between 13.0 and 32.6 s, depending on the study [5].

Several authors have already proposed ways to improve TUG by adding inertial sensors to measure acceleration or velocity, by combining it with other clinical tests, or by using cameras [7,8,9,10]. The corresponding tests are often referred to as instrumented TUG (iTUG). Their predictive performances can be correlated with a gold-standard but more complex functional tests such as Community Balance and Mobility Scale [11]. Among these approaches, we think that the addition of wearable inertial measurement units (IMUs, or inertial sensors) is particularly promising. The information contained in the measured time series (acceleration and angular velocity) goes well beyond the total time measured in TUG, making the iTUG a clinical tool that allows detailed analyses of the TUG’s different subtasks. The use of a single IMU is actually adequate to separate the TUG subtasks with sufficient accuracy for clinical applications [10,12,13]. From a methodological point of view, splitting TUG into different subtasks can improve its discriminatory power in various conditions: obese women [14], children with traumatic brain injury [15], and adults with vestibular hypofunction [16].

Using a single IMU placed on a patient’s lower back, Buisseret et al. proposed to combine the kinematic data of a 6-min walk test (6MWT) and the result of TUG to improve its predictive accuracy [7]. However, kinematic data of the TUG collected during the latter study were not analysed, despite evidence that a movement such as a trunk rotation is an index of balance measurement that requires special attention [8,11,17,18,19,20,21]. Moreover, among fall-related fractures, hip fractures are the most common. A fall during a turn increases the risk of hip fracture 8-fold compared to a fall in a straight line [18]. Therefore, in this study, we propose a detailed analysis of the two half-turns during TUG from a kinematic point of view by adding to the conventional measures such as duration and maximum angular velocity a parameter called “jerk”, which is a measure of movement’s smoothness [22].

The aim of this study is to determine: (1) which general characteristics and kinematic parameters are relevant to discriminate fallers (F) from non-fallers (NF) in a population of elderly nursing home residents, based on a dataset previously collected in [7]; (2) which of the relevant variables are best suited to predict a fall within six months using a logistic regression-based model—the model will be called i + TUG in the following to differentiate it from previous attempts called iTUG—and (3) whether i + TUG improves the predictive power of the TUG by assessing its predictive properties (sensitivity, specificity, and overall accuracy). Since our methodology seems to be very similar to the [7] proposal, it is worthwhile to outline here the difference between the present work and the latter. In [7], the duration of TUG was measured and supplemented by kinematic data from a 6MWT. A prediction of fall risk was proposed as a decision process that depends on thresholds for TUG duration and parameters that assess the variability of walk during the 6MWT. Here, fall risk is predicted only from TUG (duration and kinematic data) using multiple logistic regressions. Thus, the proposed assessment of fall risk is intended to be much shorter, with a decision criterion that can be systematically improved.

## 2. Materials and Methods

### 2.1. Population

All residents who participated in this study were at least 65 years old and lived in four different nursing homes in the Charleroi region (Belgium). All residents or their legal representatives gave their consent to participate in the study after being informed about the modalities of the study and the possible side effects. The experimental protocol is in accordance with the Declaration of Helsinki on Medical Research Involving Human Subjects and was approved by the Academic Bioethics Committee (reference B200-2017-144). The study was longitudinal and included two evaluations 6 months apart: the first one in May 2018 and the second one in November 2018. Here, we analyze data previously collected, part of which has already been analyzed in [7]. No new measurements were taken and the processed data had not been analyzed in previous studies.

The only inclusion criterion was that residents were at least 65 years old. Residents with lower limb movement disorders that prevented them from walking, cognitive disorders that prevented them from understanding the instructions given during the experiment, or cardio-respiratory disorders that prevented them from walking for 6 min were excluded. Some residents who were originally included in the sample could not be reexamined and were therefore also excluded if: they had dropped out of the study or had been hospitalized during the study period; they had one or more medical conditions that occurred between the two measurements; their medication had changed in a way that affected the measurement; and they were no longer alive.

According to all these criteria, 73 residents took part in the study until the end, giving an initial total of 92 residents. A summary of our resident’s general characteristics can be found in Table 1. Cognitive status was assessed with Hodkinson Abbreviated Mental test score (AMTS) [23] that is included in Part 1 of the Fall Risk Assessment Tool [24]. An AMTS score (on 10) ≥ 9 was considered as an intact status [24] and <7 as a possibility of dementia [25]. Residents with a possibility of dementia or diagnosed with Alzheimer’s disease were not excluded. The number of residents in faller and non-faller groups with these conditions are reported in Table 1.

### 2.2. Protocol

This study was conducted in two phases. An initial measurement, conducted in May 2018 ($t_{1}$), included: (1) a collection of information about each resident, such as medications, presence and type of a walking aid, medical history (fracture, prosthesis, disease, …); (2) the placement of a DYSKIMOT inertial sensor [7] in the back of each subject at the level of the fourth lumbar vertebra; and (3) a TUG and a 6MWT performed by each resident. The latter test is not taken into account in the present study. TUG data recording began when the “Go” instruction was given and stopped when the participant sat again on the chair. Hence, total TUG time ($T_{TUG}$) was directly measured from the length of the time series. After this initial measurement, nursing home staff were asked to record resident falls over a 6-month period. In November 2018 ($t_{2}$), based on data collected by on-site medical staff, a fall survey was conducted on each resident and they were classified as faller (F) or non-faller (NF). Nursing staff were regularly reminded to record residents’ falls through several telephone contacts.

The DYSKIMOT sensor and its placement have been discussed in detail in [7], to which we refer the interested reader. Here, we summarize some key points for completeness. The DYSKIMOT sensor (3 cm × 3 cm, 10.44 g) is based on the commercially available IMU (LSM9DS1, SparkFun Electronics, Niwot, CO, USA), which integrates a triaxial accelerometer, a gyroscope, a magnetometer and a thermometer. The IMU was attached to the resident’s back at the level of the fourth lumbar vertebra using an elastic strap. The measured time series are the three components of acceleration, $\vec{a}\left(t\right)$, and angular velocity, $\vec{\omega }\left(t\right)$, in the sensor’s frame with a sample frequency of 100 Hz. Time series were recorded on a computer via the DYSKIMOT software (v. 2.1). The sensor was placed such that the three axes of its frame correspond to the anterio-posterior (AP), medio-lateral (ML) and vertical (*V*) directions when the resident is standing at rest.

**Figure 1:**
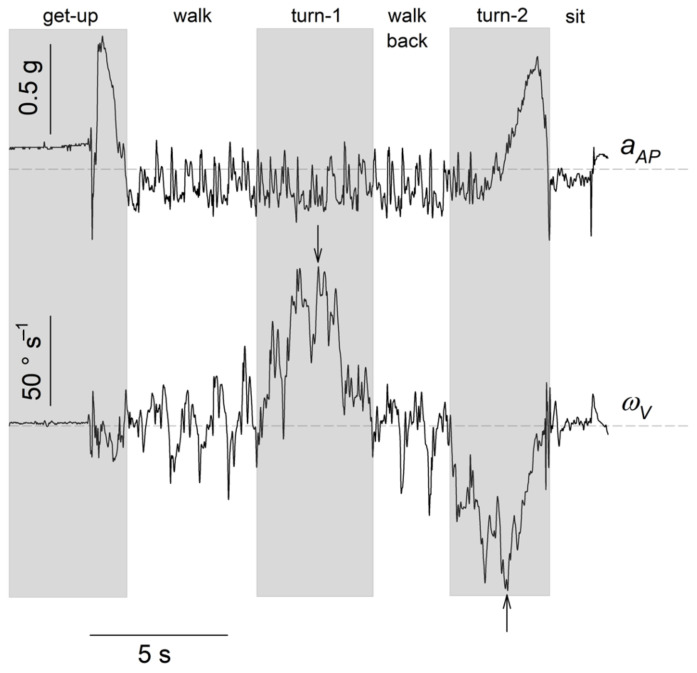
Typical traces of the AP acceleration ($a_{AP}$) and of the *V* angular velocity ($\omega_{V}$) during TUG. Acceleration is expressed in a fraction of g=9.81 m s${}^{-2}$, and angular velocity is expressed in ° s${}^{-1}$. White/gray areas highlight the different subtasks of the TUG. Arrows indicate the peak angular velocity during the two half-turns. Horizontal grey dashed lines show zero value.

Our data analysis was based on $a_{AP}$, giving the acceleration in the walking direction, and on $\omega_{V}$, giving rotation velocity during the two half-turns. These two time series contained the clearest signal in all residents and were used to determine the different subtasks of TUG. Typical traces of the AP acceleration ($a_{AP}$) and of the *V* angular velocity ($\omega_{V}$) during TUG are shown in Figure 1.

### 2.3. Division of the TUG into Subtasks and Selected Kinematic Parameters

For each resident, we divided the TUG into 6 subtasks by visual inspection of $a_{AP}\left(t\right)$ and $\omega_{V}\left(t\right)$, as illustrated in Figure 1: (1) the get-up phase occurred between the beginning of the TUG and the end of the $a_{AP}$ peak (i.e., when $a_{AP}$ comes back to a 0 value after the peak); (2) the walk phase, where $\omega_{V}$ has an oscillatory behaviour around 0; (3) the first half-turn, corresponding to the first peak in $\omega_{V}$, i.e., when $\omega_{V}$ stops oscillating around 0 to exhibit a global positive or negative trend; (4) the walk back phase, identified as the first one; (5) the second half-turn, identified as the first one; (6) the sit phase, until the end of the time series. A sharp peak in $a_{AP}$ is observed in this phase when the resident’s back hits the chair back.

After identification, the durations of the subtasks were recorded: $T_{get-up}$, $T_{walk}$, $T_{turn-1}$, $T_{walk\;back}$, $T_{turn-2}$ and $T_{sit}$. Then, a more detailed assessment of half-turns was realised, since it is known to be strongly related to participant’s stability [18]. Maximal angular velocities (in absolute values) were recorded during the first, $\omega_{V1}^{max}$, and second half-turns, $\omega_{V2}^{max}$ (Figure 1). Finally, dimensionless jerks were calculated during the first, $J_{1}$, and second, $J_{2}$, half-turns as follows [22]:

(1) $$J_{i}=\ln\left(\frac{T_{turn-i}^{3}}{\omega_{Vi}^{max\;2}}\int_{a_{i}}^{b_{i}}j^{2}\left(t\right)\;dt\;\right),$$

where $j=\frac{d\omega_{V}}{dt}$, i=1,2, and where $a_{i}$, $b_{i}$ are the time values giving the beginning and end of half-turn *i*, respectively. The derivative was computed by finite differentiation. We recall that dimensionless jerk is a measure of motion’s smoothness. The smaller the $J_{i}$, the smoother the motion. We hypothesized that NF would reach smaller durations and jerks, and larger maximal angular velocities than F.

### 2.4. Statistical Analysis

First, we evaluated the differences between the F and the NF groups, with significance level α of 0.05. For this purpose, *t*-tests were used for continuous variables, Mann–Whitney tests were used for ordinal variables (scores), and exact Fisher tests were used for categorical variables.

Second, based on the results of the above analyses, models predicting falls in our population were designed by resorting to multiple logistic regression. The logistic regression model is given by the following equation:

(2) $$\ln\left(\frac{P}{1-P}\right)=\beta_{0}+\sum_{j=1}^{n}\beta_{j}X_{j},$$

where $X_{j}$ are the *n* selected parameters, and where $\beta_{0}$, $\beta_{j}$ are fitted on the collected data via multiple logistic regression. Once the $\beta_{j}$ are fitted on the data, Equation (2) becomes a classification tool: given a set of parameters $X_{i}$, measured on one participant, the output *P* leads either to the value 0 (no predicted fall) or 1 (predicted fall). The model prediction, i.e., fall or no fall, were then compared with the actual falls of the participants. Note that several models were actually used, differing in the number of selected parameters, *n*, see below. Model performance was measured by computing sensitivity, $Se=\frac{\mathrm{True}\;\mathrm{positive}}{\mathrm{False}\;\mathrm{negative}\;+\;\mathrm{True}\;\mathrm{positive}}$, specificity, $Sp=\frac{\mathrm{True}\;\mathrm{negative}}{\mathrm{False}\;\mathrm{positive}\;+\;\mathrm{True}\;\mathrm{negative}}$ and accuracy, $Acc=\frac{\mathrm{True}\;\mathrm{positive}\;+\;\mathrm{True}\;\mathrm{negative}}{\mathrm{Total}}$. Five models (Mi with i between 0 to 4) were built for different parameter selections:

- (M0) only $X_{1}=T_{TUG}$ parameter used when comparing F and NF groups (TUG in Table 2);
- (M1) all parameters with p<0.05 used when comparing F and NF groups (kinTUG in Table 2): $X_{1}=\omega_{V1}^{max}$;
- (M2) all parameters with p<0.1 used when comparing F and NF groups (iTUG in Table 2): $X_{1}=\omega_{V1}^{max}$ and $X_{2}$ = $T_{TUG}$;
- (M3) all parameters with p<0.2 used when comparing F and NF groups (i + TUG in Table 2): $X_{1}=\omega_{V1}^{max}$, $X_{2}=T_{TUG}$, $X_{3}$ = Walking aid required (Yes = 1, No = 0) and $X_{4}$ = Gender (M = 1, F = 0);
- (M4) all parameters with p<0.3 when comparing F and NF groups (i + TUG2 in Table 2): $X_{1}=\omega_{V1}^{max}$, $X_{2}$ = $T_{TUG}$, $X_{3}$ = Walking aid required, $X_{4}$ = Gender, $X_{5}$ = $T_{turn-1}$ and $X_{6}$ = $J_{1}$.

Age was not included in our models because of its large *p*-value. It therefore has no ability to discriminate between F and NF in our sample, although a positive correlation between age and $T_{TUG}$ has been found in recent works [26,27].

*t*-tests, Mann–Whitney tests, exact Fisher tests and multiple logistic regressions were performed using SigmaPlot software (v. 14.0, Systat Software, San Jose, CA, USA).

## 3. Results

### 3.1. Population

The general characteristics of the residents are presented in Table 1. The ratio of females to males is 3 to 1 in the F group, compared with 1.2 to 1 in the NF group. The two groups did not differ significantly in any of the recorded parameters. $T_{TUG}$ is higher in F than NF as expected, with a *p*-value under the M2-threshold (Table 2). Walking aid and gender reached *p*-values below the M3-threshold (Table 2).

### 3.2. F versus NF Comparison

The comparison results for kinematic parameters are presented in Table 3. $\omega_{V1}^{max}$ was significantly different in both groups, with a higher mean value in NF. The same trend is observed for $\omega_{V2}^{max}$ but with a non-significant *p*-value. Only $\omega_{V1}^{max}$ has a *p*-value below the M1-threshold (Table 2). $J_{2}$ and $T_{turn-1}$ can be included in M4 (Table 2), while the other parameters will not be further considered.

### 3.3. Multiple Logistic Regressions

Results from the multiple logistic regressions are shown in Table 2. It is readily observed that M3 (i + TUG) reaches the best performances (grey area), and that adding extra parameters (M4) does not improve M3.

## 4. Discussion

Our clinical challenge was to improve the predictive ability of the well-known TUG in two ways: (1) by instrumenting it to assess multiple quantitative kinematic parameters specific to the different subtasks of the TUG and (2) by including qualitative features of the residents. Our multiple logistic regressions led to the development of an i + TUG (M3-model in Table 2) for predicting fall risk in our sample that included the parameters of: a TUG with $T_{TUG}$, an iTUG with $\omega_{V1}^{max}$, and walking aid and gender characteristics.

Thirty-six residents used walking aids to compensate for postural instability and/or mobility decline (F = 11 and NF = 26). In our sample, the postural instability and/or mobility decline typically have several causes, which include diabetic polyneuropathy (F = 3 and NF = 8), Alzheimer’s disease (F = 5 and NF = 14), and post-stroke hemiparesis (F = 2 and NF = 4). No residents with Parkinson’s disease were included, but this condition was not an exclusion criteria.

The predictive ability of a fall risk test is a critical component of evidence-based patient care, especially among elderly nursing home residents. The indicators of predictive performance we obtained for i + TUG are better than those for TUG, which shows how interesting it is to add additional information to TUG. Our model can be compared with previously proposed models. In [28], the limited predictive ability of TUG for identifying F in a sample of community-dwelling older adults was already pointed out. They showed a sensitivity of 32% (versus 29.2% with our i + TUG) and a specificity of 73% (95.9%).

In a preliminary study conducted with the same sample of residents [7], we improved the discriminative and predictive qualities of TUG by adding kinematic data collected during a 6MWT. The addition of kinematic factors increased the accuracy of the test from 65.7% to 73.9%, with a sensitivity of 85% and a specificity of 50%. Here, we have shown that, based on data collected with the same sensor in the same sample, it is possible to achieve the same predictive accuracy using only data collected during i + TUG. Combining the results of our previous study [7] and the findings obtained here, it appears that an instrumented 6MWT (i6MWT) and the i + TUG developed here are highly complementary, with one test having a high sensitivity and the other high specificity. Because sensitivity refers to the test’s potential to identify F residents and specificity refers to the test’s potential to identify NF residents, we recommend that, in daily clinical practice and long-term monitoring of nursing home residents, each resident should undergo an i + TUG in the first instance to rule out that he/she is at risk for falls. If the i + TUG can not rule out that he/she is at risk for falls, an i6MWT must also be performed to confirm that he/she is indeed at risk.

The mean $T_{TUG}$ was 24.5 s for F and 21.5 s for NF, both values that are close to the 22.5 s found in [4] and the 22.1 s found in [29]. Furthermore, the inclusion of kinematic data from a single wearable IMU sensor allowed for computing TUG’s subtask durations. It appears that our mean $T_{get-up}$ (4.3 ± 2.7 s) is higher than the value reported in [9] (2.1 ± 0.3 s). This discrepancy can be explained by the fact that the get-up movement is first initiated by a trunk tilt ($\omega_{ML}$, not studied here) and terminated by a hip extension ($a_{AP}$). We chose the latter way of identifying the get-up phase, while, in [9], they chose the former. Our larger value can also be explained by the age of our sample. As shown in [30], there is indeed a relationship between sarcopenia in the elderly and the long time it takes them to get up.

A clear finding in our study is that $\omega_{V1}^{max}$ is significantly higher in NF. This result is consistent with previous findings. In the study [18] that led to the development of the “Dite Turn test”, it was shown that elderly people who are more prone to fall turn more slowly and unsteadily. They highlighted four characteristics of the turn performed in the TUG that distinguish F from NF: the time required to turn, i.e., $T_{turn-i}$ that we found to be higher in F, but also indirectly $\omega_{Vi}^{max}$; the number of steps to complete the turn; the stable appearance of the subject during the turn and the fact that the subject makes a smooth transition between the turning and walking subtasks. The number of steps was not measured in this work. However, the smoothness/stable appearance of the movement was evaluated using $J_{i}$. We found that $J_{i}$ is decreased in NF, i.e., their motion is smoother in agreement with the observation of [18], although the difference is not significant. In [19,20], it is also highlighted that the duration and velocity of rotation measured by an inertial sensor during a 7-day period were increased in F. The i + TUG is able to reach the same conclusion within a much shorter time period. Same conclusions about $\omega_{Vi}^{max}$ have been found in [19]. As for our results, the jerk was measured during the first and second half-turns. It would have been interesting to extend the measurement to the end and the beginning of walking in the case of the first half-turn, and to the end of walking and the beginning of the transition to the sitting position in the case of the second half-turn. Indeed, it was shown in [13] that the strategies for approaching the half-turn differ between young and old participants regarding velocity. Interestingly, $\omega_{V2}^{max}$ is not significantly different between F and NF. This could be due to greater variability in our sample. As pointed out in [31], the second half-turn requires more cognitive skills, different motor planning, and greater visual skills because of the need to anticipate the sitting phase.

We acknowledge that our sample size may be considered small for a geriatric population, which is a limitation of our study. In addition, our measurements were performed in nursing homes and may not be representative of the majority of the elderly population. TUG assesses only the person’s global mobility; other risk factors such as visual or cognitive impairment, or polymedication [5] need to be considered to capture the entire clinical picture. To address this limitation, it would be interesting to combine the results of the i + TUG with other tests, such as the Falls Risk Assessment Tool (FRAT), which takes into account the other risk factors mentioned above. Our sample was not designed to examine age effects on fall risk because both F and NF have similar means and a large range of ages. However, age increases $T_{TUG}$ [27], and presumably the fall risk. A review paper [32] found that people aged 85 years or older in the United States are four times more likely to be injured in falls than a population aged 65 to 74 years. Lower limb weakness and, more generally, sarcopenia may be partly responsible for this [33,34]. Knee extensor muscle strength was not assessed. Since knee extensor muscle strength could identify the elderly at risk of falling [35], these missing data are also a limitation of our study. Note that the threshold of 85-years in the review [32] is close to the mean age of our sample, illustrating the importance of predicting fall risk in very old people. A wider spread of age would be required, which is an interesting perspective. Finally, we excluded residents with cognitive disorders that prevented them from understanding the instructions given during the experiment. However, even a mild cognitive impairment could affect the iTUG subtasks [31]. Our protocol does not allow us to determine the impact of mild cognitive impairment on our findings.

## 5. Conclusions

We have shown that integrating kinematic data collected with a single low-cost IMU during TUG and general resident characteristics can improve the accuracy of fall risk prediction. The new i + TUG achieves 74% versus 67% for the TUG. The i + TUG is highly specific (95.9%) and quick to perform; it may be implemented on a smartphone. We recommend integrating the i + TUG into the test battery commonly performed in nursing homes to rule out residents at risk for falls with a high degree of confidence.

It should be kept in mind that the management of an elderly resident in a nursing home must be multifactorial. The i + TUG must therefore be integrated to optimize a care and diagnostic approach that does not neglect the psychosocial and behavioural aspects and always focuses on the resident/caregiver duo.

## Figures and Tables

**Table 1:** General characteristics of the residents. Fallers were identified according to the fall records between the 6-months interval ($t_{1}$ and $t_{2}$). Numerical data are written under the form mean ± standard deviation (*t*-test performed) or median [Q1–Q3] (Mann–Whitney test performed). For the age, the minimum and maximum values in each group are given (second line). Exact Fisher tests were performed for the categorical data (M/F or Yes/No). *p*-values for the comparison between fallers (F) and non-fallers (NF) groups are given in the last column. Total TUG time ($T_{TUG}$) was assessed with a stopwatch. Medications included: psychotrope, antiarrhythmic, and diuretics. Hypertension is defined as a value >140/90 mmHg.

Parameter	F	NF	*p*
Residents (*n*)	24	49	
Age (years)	84±9	83±8	0.646
	66–96	65–96	
Medication	4 [2–5]	3 [2–5]	
FRAT	11 [10–14]	10 [8–12]	
$T_{TUG}$ (s)	24.5 ± 8.5	21.5 ± 8.1	0.096
Gender (M/F)	6/18	22/27	0.128
Walking aid required (Yes/No)	15/9	21/28	0.140
Post-stroke hemiparesis (Yes/No)	2/21	4/45	0.677
Possibility of dementia (Yes/No)	3/21	9/40	0.739
Alzheimer disease (Yes/No)	5/19	14/35	0.577
Previous heart surgery (Yes/No)	8/16	9/40	0.238
Diabetic polyneuropathy (Yes/No)	3/21	8/41	1
Hip or knee replacement (Yes/No)	3/21	9/40	0.739

**Table 2:** First seven rows: $\beta_{i}$ coefficients fitted from model (2). The first row gives $\beta_{0}$. Last three rows: performance indicators of the models (Se: sensitivity, Sp: specificity, Acc: accuracy).

Parameters	M0	M1	M2	M3	M4
$X_{i}$	(TUG)	(kinTUG)	(iTUG)	(i + TUG)	(i + TUG2)
$\beta_{0}$	−1.709	1.423	0.822	1.207	1.553
$\omega_{V1}^{max}$		−0.0245	−0.0213	−0.0208	−0.0231
$T_{TUG}$	0.0434		0.0139	−0.0046	0.0197
Walking aid required				0.403	0.333
Gender				−0.637	−0.649
$J_{1}$					−0.0027
$T_{turn-1}$					− 0.124
Performance Indicators					
Se (%)	8.3	8.3	12.5	29.2	20.8
Sp (%)	95.9	91.8	91.8	95.9	91.8
Acc (%)	67.1	64.4	65.7	74.0	68.5

**Table 3:** Comparison of kinematic parameters between fallers (F) and non-fallers (NF) groups. Data are written under the form mean ± standard deviation. *p*-values for the comparison between F and NF groups are given in the last column; parameters are ordered by increasing *p*-values, with significant values in bold font.

Parameters $X_{i}$	F	NF	*p*
$\omega_{V1}^{max}$ (° s${}^{-1}$)	82.0±19.0	92.9±23.4	**0.031**
$J_{1}$	12.0±2.3	11.6±2.8	0.203
$T_{turn-1}$ (s)	5.2±2.2	4.9±2.5	0.293
$J_{2}$	12.0±3.2	11.4±3.9	0.304
$\omega_{V2}^{max}$ (° s${}^{-1}$)	96.3±24.5	106.2±35.0	0.315
$T_{get-up}$ (s)	4.8±3.5	4.0±2.2	0.338
$T_{sit}$ (s)	3.7±3.4	3.1±1.9	0.545
$T_{turn-2}$ (s)	4.3±2.3	3.8±2.0	0.569
$T_{walk}$ (s)	4.2±2.2	4.0±3.0	0.634
$T_{walk\;back}$ (s)	3.9±2.7	4.0±4.1	0.773

## Data Availability

Data are available here: https://osf.io/q45ny/ (accessed on 18 January 2022). In particular, the file Analysed_Data.xlsx contains the parameters $X_{i}$ needed to fit the $\beta_{i}$ coefficients according to Equation (2).
